# Sex-related differences in clinical characteristics of children with ASD without ID: Results from the ELENA cohort

**DOI:** 10.3389/fpsyt.2022.998195

**Published:** 2022-11-28

**Authors:** Florine Dellapiazza, Cécile Michelon, Cécile Rattaz, Marie-Christine Picot, Amaria Baghdadli

**Affiliations:** ^1^Centre de Ressources Autisme Languedoc-Roussillon et Centre d’Excellence sur l’Autisme et les Troubles Neurodéveloppement (CeAND), CHU Montpellier, Montpellier, France; ^2^Department of Medical Information, University Hospital, Montpellier, France; ^3^UVSQ, Inserm, CESP, Team DevPsy, Université Paris-Saclay, Paris, France; ^4^Faculté de Médecine, Université de Montpellier, Montpellier, France

**Keywords:** autism spectrum disorder, sex ratio, preschooler, SRS-2, cohort study

## Abstract

**Objective:**

The literature on sex related-clinical differences for children with autism spectrum disorder (ASD) is highly contradictory, whereas this topic has major clinical implications. We aimed to investigate sex-related clinical differences in children with ASD without intellectual disability (ID).

**Materials and methods:**

We compared 319 boys and 65 girls with ASD without ID, aged from 2 to 12 years, recruited from a multiregional cohort on their clinical profiles based on the scores for the Vineland-II, the SRS-2, the ADOS calibrated severity score, sensory processing, aberrant behaviors, and comorbidity rates.

**Results:**

Our results confirm a high sex ratio of 4.9 males/females. Many similarities were found in the clinical profiles. However, we found that girls had higher SRS-2 total scores. In addition, there was a negative correlation between the SRS-2 total score and the intellectual quotient level (IQ) for girls only.

**Conclusion:**

We confirm the higher rates of boys with ASD without ID. A comparison between the girls and boys showed them to have similar clinical profiles, except for the SRS- 2 total scores, which were higher among girls, suggesting more severe social impairment perceived by parents. Our findings that the cognitive level is related to ASD severity in girls should be taken into account during the diagnostic procedure in the clinical interpretation of gold-standard measures of ASD, and additional clinical observations are necessary.

**Clinical trial registration:**

[ClinicalTrials.gov], identifier [NCT02625116s].

## Introduction

Autism spectrum disorder (ASD) is an early and chronic neurodevelopmental disorder for which the estimated prevalence has dramatically increased over the last five decades, with rates that remain higher among males (1). In the past ASD was thought to be a rare condition, affecting about one in 2,000 individuals while the Centers for Disease Control and Prevention (CDC) in United States estimated in 2021 the prevalence at 1 in 44 children (2, 3). In this heterogeneous condition, the sex ratio differs according to the child’s clinical characteristics, such as the severity of ASD symptomatology, intellectual level, or chronological age (4). Indeed, although the overall sex ratio in ASD is estimated to be approximately 4:1, it is 2:1 in cases of associated intellectual disability (ID) and 11:1 in the absence of ID (3, 5–7). Moreover, the sex ratio is estimated to be 5:1 during childhood and 2:1 during adulthood (4, 8, 9).

Several explanations have been proposed to account for the sex ratio in ASD. First, it was suggested that the preponderance of boys with a diagnosis of ASD was due to a gender-related vulnerability determined by genetic and hormonal factors potentially involved in the onset of ASD (10, 11). Second, a number of studies have claimed the existence of a typical “female” phenotype elicited by sex-related differences in clinical symptoms of ASD (12). Thus, girls with ASD may be under-identified in clinical samples because they were missed during the diagnostic process and/or received alternative diagnoses, such as anxiety disorder or depression (13).

In the recent literature on the association between sex and ASD severity, two studies using gold-standard measures of ASD, including the Autism Diagnostic Observation Schedule, second edition (ADOS-2) and the Social Responsiveness Scale (SRS-2), found similar profiles for both sexes (14, 15). However, Rodgers et al. (15) showed on a small sample of 34 girls matched on IQ and chronological age with 34 boys that the SRS-2 score negatively correlated with the intelligence quotient (IQ) in girls with ASD. Consistent with this observation, another study found in a larger sample of 114 girls matched on IQ and age with 114 boys, that ASD was less often identified among high functioning girls (IQ > 70) using the Autism Diagnostic Interview (ADI) than among boys with a comparable IQ (16). These conflicting results suggest that more studies using ASD diagnostic gold standard measures are needed to confirm sex-related differences in the clinical presentation of ASD.

A meta-analysis of 22 studies published before 2014 examining sex-related differences in socialization and communication skills of individuals with ASD across five age categories from toddlerhood to young adulthood suggested similar profiles between males and females (17). However, a study published in 2019 suggested that girls with ASD were more socially motivated by friendships at all ages, with the limitation that this study was not a systematic review (18). Another recent study of 54 girls with a diagnosis of ASD aged 3–18 years matched on age and cognitive level with 55 boys also confirmed better social skills in girls than in boys during childhood (19). In contrast, a study in a large sample of adults with a diagnosis of ASD involving 304 girls and 2,114 boys found that females had poorer social and communication cognitions (8).

Concerning repetitive and restricted behaviors (RRB), two studies using RRB and SRS-2 scales found similar sex-related clinical patterns (16, 20). Two other studies found that the severity of RRB assessed by the ADOS was greater in boys in adults (8), and in a large sample of 1024 participants aged 2–12 years (21) and a literature review concluded that boys had more RRB (18). However, a meta-analysis of longitudinal studies published before 2014 found early sex-related differences in RRB in childhood up to age six, but not later (17). A recent study that compared 26 girls and 142 boys with ASD focusing on sensory processing showed sex-related differences, with girls showing greater auditory and/or vestibular impairment (22).

Two studies showed similar IQ levels, regardless of sex during childhood (21, 23). However, a recent literature review of sex-related differences in IQ among individuals with ASD from pre-school age to adulthood confirmed similar executive function abilities during childhood but emerging differences from adolescence (18). By contrast, a recent study comparing 54 females with ASD to a group of 55 males with ASD, both without ID, matched for age, reported a higher verbal IQ for girls (19).

In terms of adaptive skills measured by the Vineland Adaptive Behavior Scales version II (VABS-II), one study that compared 114 school-aged girls with ASD matched for IQ and age with 114 boys did not find any sex-related differences (16). By contrast, another study of 115 children with ASD aged from 7 to 13 years (24) found that adaptive skills measured using the adaptive behavior assessment system (ABAS) (25) scale were weaker for girls. One study showed that the gap between IQ and socialization skills on the VABS-II was higher for girls than boys with ASD between the ages of 8 and 17 years, in contrast to those with typical development (26).

Concerning comorbidities, one study found similar rates of attention deficit hyperactivity disorder and anxiety disorders among 54 females with ASD and a group of 55 age-matched males with ASD, both without ID (19). Another study found similar levels of internalizing and externalizing behaviors assessed by the Behavior Assessment System for Children, (BASC2 PRS) (27) among 40 girls and 40 boys with ASD, both without ID, matched for age and IQ (28). Conversely, using the Child Behavior Checklist (CBCL), one study showed higher scores of internalizing behaviors among boys of preschool age with ASD (14) and another study, higher scores of externalizing behaviors for women with ASD during adulthood (8).

To date, the literature on sex related-clinical differences for children with ASD is highly contradictory, whereas this topic has major clinical implications for tailoring the detection, early diagnosis, and interventions of ASD for both males and females, as it has been suggested that females may be under or mis-diagnosed. The goal of this study was to investigate sex-related clinical differences in a large cohort of children with ASD. Our sample targeted children with ASD without ID to aid comparisons with previous studies, which mainly focused (14, 15, 19, 28) on children without ID.

In this study, we use the term “sex” to refer to “a person’s biological status,” which is different from “gender.”

## Materials and methods

### Participants

**FIGURE 1:**
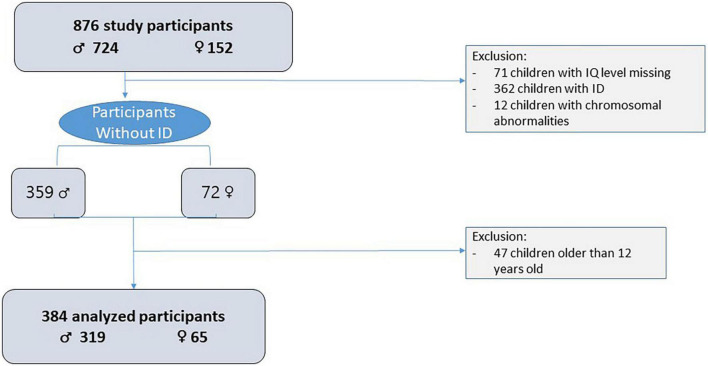
Selection of participants in the ELENA cohort.

Participants in our sample of children with ASD and without ID were recruited from the ELENA cohort (Longitudinal Study of Children with Autism), an ongoing prospective and multiregional cohort of children with ASD (29). In total, 876 children were included in the ELENA cohort between 2013 and 2019 according to their diagnosis, age (2–16 years), and parental consent. All children received a clinical diagnosis of ASD according to the DSM-5 criteria, confirmed by a multidisciplinary team using a standardized process, including the ADOS-2 and the ADI-Revised (ADI-R), administered by licensed and trained psychologists, a parental interview about the child’s adaptive functioning using the VABS-II, and direct psychological examinations to assess cognitive level using appropriate psychometric test according to age [Wechsler scales (30–33) or K-ABC-II scale (34)]. For this study, the selection criteria were children recruited from the ELENA cohort having no ID (IQ > 70) and aged from 2 to 11 years and 11 months. The exclusion criterion was children with chromosomal abnormalities. The selection process is described in Figure 1. The sample included 384 participants aged in mean of 6.3 years (SD = 2.7 years) with a male/female sex ratio of 4.9.

### Measures

Caregivers completed questionnaires electronically on a web database, including the CBCL, SRS-2, and Aberrant Behavior Checklist (ABC).

ASD symptomatology was assessed using the SRS-2, a 65-item questionnaire measuring ASD trait severity (35). For the present study, we used the school-age form (from 2.5 to 18 years), which assesses social impairment across two principal subscales: Restricted Interests and Behavior (RIRB) and Social Communication and Interaction (SCI). SCI consists of 4 subscales, including social awareness, social cognition, social communication, and social motivation.

Each question was rated from 1 (not true) to 4 (almost always true). Raw scores converted to standardized T-Scores (*M* = 50, SD = 10) were used to assess the severity of symptoms. Total scores of ≤59 correspond to the normal threshold, 60–65, a mild degree of impairment, 66–75, a moderate degree of impairment, and 76 or higher, a severe degree of impairment. The internal consistency was 0.95.

*ASD symptom severity* was examined using the ADOS-2 (36), a semi-structured behavioral observation protocol that assesses ASD symptomatology. We used the Calibrate Severity Score (CSS), ranging from 1 to 10, a higher score corresponding to greater ASD severity. The internal consistency ranged from poor to excellent (α = 0.50–0.92), test-retest reliability was acceptable (0.64–0.88), and interrater reliability ranged from good to excellent (0.79–0.98).

*ASD symptoms* were also assessed through the ADI-R, a semi-structured interview administered to parents (37). The three algorithm scores for the communication, social reciprocity and restricted and repetitive behaviors domains were studied.

*Sensory processing* was assessed using the Sensory Profile questionnaire. A total score of the Sensory Profile can be calculated from 38 items extracted from the long version (38). Lower scores indicate greater sensory processing difficulties. Internal consistency ranged from 0.70 to 0.90 and internal validity correlations from 0.25 to 0.76.

*Intellectual functioning* was assessed from psychometric scales, depending on the age of each participant. The non-verbal cognitive level was estimated from the “fluid reasoning” dimension of the WISC-V (30) and the WPPSI-IV (31), “perceptual reasoning” dimension of the WISC-IV (32), and the “performance IQ” of the WPPSI-III (33) or “simultaneous process” of the K-ABC II (34).

*Adaptive functioning* was assessed using the VABS-II (39). This standardized caregiver interview of 297 items measures adaptive behaviors in the subdomains of communication, daily living skills, and socialization. The reliability of the VABS-II for each domain was excellent (α = 0.80) and the intra-class coefficient of the test/re-test 0.89.

*Aberrant behaviors* were assessed using the ABC (40), a 58-item scale concerning problem behaviors, with each item scored from 0 (no problem) to 3 (severe). The scale includes four factors: I) irritability, agitation, crying; II) lethargy, social withdrawal; III) stereotypic behavior; and IV) hyperactivity, non-compliance. The ABC showed good internal consistency (α = 0.91), excellent test–retest reliability of 0.98, and an acceptable interrater reliability of 0.63.

*Externalizing and internalizing behavioral* problems were assessed using the CBCL (41), a standardized caregiver-report exploring emotional and behavioral problems in children and adolescents aged from 2 to 18 years. The CBCL provides two scales of child behavior: internalizing and externalizing problems. T-scores based on age and sex were obtained; T-scores ≥ 70 are considered clinically significant and T-scores from 65 to 69 borderline clinically significant. Internal consistency ranged from 0.72 to 0.91 and the inter-rater reliability from 0.63 to 0.88. The test-reliability was 0.90.

Data concerning the children’s ages at referral and first diagnosis, as well as data on attendance of mainstream schools (yes/no) or a specialized setting (yes/no) and comorbidities were collected through a parental self-reported questionnaire.

### Statistical analysis

We determined the male to female ratio (MFR) for all ASD and then divided all ASD into participants with and those without ID. Only participants without ID and aged from 2 to 11 years and 11 months at inclusion were analyzed, because the group aged more than 12 years was too small. Independent sample *t*-tests, Mann–Whitney–Wilcoxon tests, or Chi square tests were performed to evaluate sex-related differences in ASD symptoms, intellectual functioning, and adaptive skills variables [i.e., age, IQ, and autism severity level (Table 1)] on comorbidity and aberrant behaviors (Table 2). The discrepancies in the size of the population shown in Table 2 are related to variations in the completion of self-administered questionnaires by parents.

**TABLE 1:** Sex-related differences for autism spectrum disorder symptoms, intellectual functioning, and adaptive skills (*N* = 384).

	ALL			Boys		Girls		*p*-value
	*N* = 384			*N* = 319		*N* = 65		
	*n*		Mean ± SD	*n*	Mean ± SD	*n*	Mean ± SD	
Age	384		6.3 ± 2.7	319	6.2 ± 2.7	65	6.8 ± 2.85	0.1^ε^
IQ	384		92.9 ± 15.6	319	93.5 ± 16.2	65	90.2 ± 12.5	0.3^ε^
**Education**								
Attending ordinary school (Yes)	227/252		90.1	189/209	90.4	38/43	88.4	0.8^α^
Special education or care service (Yes)	101/252		40.1	88/209	42.1	13/43	30.2	0.1^α^
**Comorbidities**								
Language disorders	34/384		8.85	30/319	9.4	4/65	6.2	0.4^α^
Attention deficit hyperactivity disorder	57/384		14.8	47/319	14.7	10/65	15.4	0.9^α^
Developmental coordination disorder	25/384		6.5	19/319	6.0	6/65	9.2	0.4^α^
Age first psychiatric advice	250		3.3 ± 1.8	207	3.3 ± 1.7	43	3.7 ± 2.3	0.3^ε^
Age first diagnosis	252		5.4 ± 2.7	209	5.3 ± 2.6	43	5.7 ± 3.2	0.6^ε^
ADOS severity score	343		6.6 ± 2.0	285	6.6 ± 1.9	58	6.4 ± 2.3	0.5^ε^
**ADI-R**								
Verbal communication		256	12.4 ± 5.2	207	12.2 ± 5.1	46	13.0 ± 5.95	0.35^γ^
Non-verbal communication		129	8.8 ± 3.9	106	9.1 ± 3.75	23	7.4 ± 4.3	0.1^ε^
Social reciprocity		343	14.8 ± 6.0	286	14.9 ± 5.9	57	14.6 ± 6.6	0.7^γ^
Restricted and repetitive behaviors	343		5.0 ± 2.5	273	3.5 ± 1.3	54	3.4 ± 1.6	0.9^ε^
**SRS-2**								
Social awareness	181		76.7 ± 14.0	145	76.1 ± 14.1	36	79.25 ± 13.6	0.3^ε^
Social cognition	169		87.7 ± 17.1	135	86.4 ± 17.0	34	92.8 ± 17.0	**0.03** ^ε^
Social communication	166		87.0 ± 17.1	133	85.7 ± 17.3	33	92.45 ± 15.6	**0.04** ^γ^
Social motivation	177		76.0 ± 16.0	142	75.8 ± 16.4	35	76.5 ± 14.4	0.8^γ^
Restricted interests and repetitive behavior	178		98.3 ± 23.6	143	94.1 ± 21.3	35	115.4 ± 25.2	**0.0001** ^γ^
Social communication and interaction	155		88.8 ± 17.1	124	87.6 ± 17.2	31	93.35 ± 16.0	**0.04** ^ε^
Total score	184		91.8 ± 18.3	148	90.2 ± 18.15	36	98.5 ± 17.8	**0.01** ^γ^
**VABS-II**								
Communication	384		78.9 ± 13.3	319	79.1 ± 13.3	65	78.2 ± 13.0	0.7^ε^
Socialization	384		74.5 ± 10.0	319	74.4 ± 10.1	65	75.2 ± 9.7	0.3^ε^
Daily living skills	384		79.2 ± 11.2	319	79.5 ± 10.8	65	77.9 ± 12.95	0.3^ε^
**Sensory profile**								
Short total score	220		135.6 ± 23.0	183	135.5 ± 23.1	37	136.2 ± 22.5	0.9^ε^

Significant differences are shown in bold. ^α^Chi-square. ^ε^Mann–Whitney–Wilcoxon test. ^γ^Student test. SD, standard deviation; VABS-II, Vineland Adaptive Behavior Scale second version; ADI-R, Autism Diagnostic Interview-Revised; SRS-2, Social Responsiveness Scale; IQ, intellectual quotient.

**TABLE 2:** Sex-related differences in comorbidities and aberrant behaviors (*N* = 384).

Other measures of ASD		ALL		Boys		Girls		*p*-value
		*n* = 384		*n* = 319		*n* = 65		
		*N*	% or Mean ± SD	*N*	% or Mean ± SD	*N*	% or Mean ± SD	
Child Behavior Checklist (CBCL)*								
Broad-brand scale	Internalizing problems	88/144	61.1	59/105	56.2	29/39	74.4	**0.04^α^**
	Externalizing problems	126/222	56.8	104/183	56.8	22/39	56.4	0.9^α^
DSM-Oriented scales	Affective problems	127/221	57.5	102/182	56.0	25/39	64.1	0.35^α^
	Anxiety problems	117/221	52.9	91/182	50.0	26/39	66.7	**0.05^α^**
	Attention deficit/ Hyperactivity problems	90/221	40.7	69/182	37.9	21/39	53.8	0.07^α^
	Oppositional defiant problems	60/222	27.0	49/183	26.8	11/39	28.2	0.8^α^
	Somatic problems**	24/124	19.4	19/96	19.8	5/28	17.9	0.8^α^
	Conduct problems**	33/124	26.6	26/96	27.1	7/28	25.0	0.8^α^
	Pervasive problems***	71/97	73.2	62/85	72.9	9/12	75.0	1^α^
**Aberrant Behavior Checklist (ABC)**				*Boys, n* = *192*		*Girls, n* = *39*		
	Irritability, uncooperative	230	32.0 ± 20.0	192	32.4 ± 20.0	38	29.9 ± 20.5	0.5^ε^
	Lethargy, withdrawal	228	23.2 ± 16.1	190	22.5 ± 15.8	38	26.5 ± 17.5	0.2^ε^
	Stereotypy	229	27.8 ± 21.9	191	27.7 ± 21.9	38	28.1 ± 22.2	0.9^ε^
	Hyperactivity	230	42.6 ± 23.2	191	42.8 ± 23.7	39	41.7 ± 21.1	0.9^ε^

Significant differences are shown in bold. *Prevalence of borderline/clinical levels of behavioral and emotional problems (BEP) are presented. **Only for children over 6 years of age. ***Only for children under 6 years of age. ^α^Chi-square. ^ε^Mann Whitney test. SD, standard deviation; CBCL, Child Behavior Checklist; ABC, Aberrant Behaviors Checklist.

Simple bivariate Pearson/Spearman correlations were used to assess the possible association between SRS-2 total score, clinical data and sex. *Post hoc* comparisons were performed using the Bonferroni test. Only differences with an adjusted *p* < 0.05 were considered statistically significant. All analysis was performed using SAS 7.3.

### Ethics

The study and informed consent procedure were approved by the Ethics Committee on the Research of Human Subjects at Marseille Mediterranean University and the National Commission for Computing and Liberties (CNIL. number DR-2015-393). All participating families signed an informed consent form. All procedures were performed in accordance with the relevant guidelines and regulations.

## Results

### Sex-related comparisons for clinical symptoms

Sex-related comparisons for ASD symptoms, IQ, and adaptive skills are presented in Table 1. There were no significant sex-related differences for age, school attendance, comorbidities, age at first diagnosis, ADOS and ADI-R scores, IQ, VABS-II score, Sensory profile, (all *p* > 0.05). The only significant sex-related differences were that girls had higher scores than boys for the total score SRS-2 (*t* = –2.5, *p* = 0.01), social cognition (*z* = 2.2, *p* = 0.03), social communication (*t* = –4.61, *p* = 0.04), SCI (*z* = 2.1, *p* = 0.04) and restricted interests and repetitive behaviors (*z* = 4.5, *p* = 0.0001).

### Sex-related comparisons for comorbidities

Sex-related comparisons for comorbidities (CBCL and ABC scores) are presented in Table 2. There were no significant sex-related differences for externalizing problems, affective problems, attention deficit/hyperactivity problems, oppositional defiant problems, somatic problems, conduct problems, pervasive problems assessed through the CBCL (all *p* > 0.05). The only significant sex-related difference was that girls had a higher score than boys for internalizing problems (*p* = 0.04) and anxiety problems (*p* = 0.05). No significant sex related differences were found regarding the ABC four domains.

### Correlations between SRS-2 scores and clinical characteristics for girls

**FIGURE 2:**
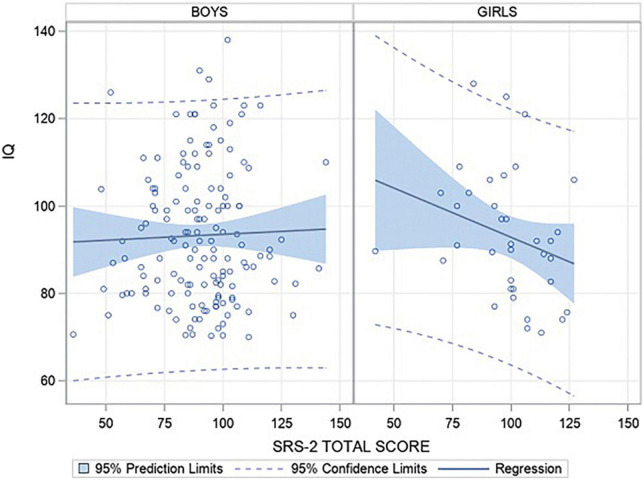
Correlation between SRS-2 total score and IQ level by sex.

Correlations that were significant only for the girls group (and not for the boys group) are presented here (see Supplementary Table 1 for the other correlations). IQ moderately negatively correlated with the SRS-2 total score (*r* = –0.37, *p* = 0.02; Figure 2), social cognition score (*r* = –0.51, *p* < 0.001), social motivation score (*r* = –0.39, *p* = 0.02), and restricted interests and repetitive behaviors score (*r* = –0.4, *p* = 0.01). Thus, when girls had higher IQ they had lower SRS-2 score (respectively, total, social cognition, motivation and restricted interests and repetitive behaviors scores).

### Correlations between SRS-2 scores and clinical characteristics for boys

Correlations that were significant only for the boys group (and not for the girls group) are described here (see Supplementary Table 2 for the other correlations). The SRS-2 total score weakly to moderately negatively correlated with the three VABS-II sub scores (communication: *r* = –0.23, *p* < 0.001, socialization: *r* = –0.40, *p* < 0.001, daily living skills: *r* = –0.20, *p* = 0.02) and with the ADI-R scores (verbal communication: *r* = 0.40, *p* < 0.001, non-verbal communication: *r* = 0.3, *p* = 0.05, social reciprocity: *r* = 0.5, *p* < 0.001, restricted and repetitive behaviors: *r* = –0.3, *p* < 0.001). Indeed, when boys had lower ASD severity assessed by SRS-2 and ADI-R they had higher adaptive skills. The SRS-2 total score moderately correlated with the hyperactivity score on the ABC scale (*r* = 0.46, *p* < 0.001). When boys had a high ASD symptoms severity according to the SRS-2 total score, they also had a high level of hyperactivity. For the CBCL score, boys in the group within the normal threshold had significantly lower SRS-2 total scores than those in the group with a score above the clinical threshold for externalizing problems (*M*_normal_ = 82.8 vs *M*_clinical_ = 95.8, *p* < 0.001), attention disorder problems (*M*_normal_ = 86.4 vs *M*_clinical_ = 96.4, *p* = 0.02), oppositional problems (*M*_normal_ = 88.0 vs *M*_clinical_ = 98.3, *p* = 0.01) pervasive development (*M*_normal_ = 66.8 vs *M*_clinical_ = 101.3, *p* < 0.001) and affective problems (*M*_normal_ = 81.8 vs *M*_clinical_ = 97.4, *p* < 0.001).

## Discussion

The current study aimed to investigate sex-related clinical differences among children with ASD and without ID using a large cohort of children from the French ELENA cohort, for which children were recruited from the age of 2 years to 11 years and 11 months. As expected, the 4.9 sex ratio in our sample confirmed a higher rate of boys than girls for individuals with ASD without ID (3, 5, 7).

Our findings also corroborate those of previous studies suggesting that girls and boys have similar clinical profiles for numerous clinical dimensions, such as adaptive skills measured with the VABS-II, ADOS, and ADI-R, ASD severity, sensory processing, aberrant behaviors, and comorbidities (14, 19, 20). However, de Giambattista et al. (19) assumed that possible subtle differences were undetectable by common screening methods, which are not sufficiently sensitive. Lawson et al. (18) added that certain differences in cognition or executive functioning emerge only from adolescence.

In our study, one sex-related clinical difference was found for ASD symptomatology measured with the SRS-2. Indeed, girls showed greater impairment than boys, suggested by both a higher total score and higher sub-scores (for social communication, social cognition, and social communication interaction). Moreover, we found a moderate positive correlation between the SRS-2 total score and IQ only among girls, suggesting that girls who had less cognitive impairment also had fewer ASD symptoms as measured with the SRS-2. As this association was not found for the ADOS score, which is a direct measure of ASD symptomatology, we hypothesize that this result is related to the use of the SRS-2 parental questionnaire involving potential parental perceptive bias due to social expectations about their daughters (42). Another possible explanation could be that higher cognitive levels in girls with ASD have an impact on their ability of “social camouflaging,” that is to say, to compensate or mask their symptoms (13).

We did not find sex-related differences in adaptive skills, as other authors (16, 26). However, our results showed a moderate correlation between the severity of ASD symptomatology assessed by the SRS-2 and adaptive level only in the boy’s group. Indeed, this result shows that higher ASD severity in boys is associated with lower adaptive skills, indicating that ASD severity tends to affect adaptive skills in boys without ID. One study showed that boys with ASD presented a gap between the level of adaptive skills and cognitive abilities, which increased with age (26). Thus, more longitudinal studies will be necessary to understand this association using a developmental approach.

Another sex-related clinical difference was found for the level of internalizing behaviors assessed using the CBCL, especially for the anxiety dimension, which was higher in girls. This difference was not expected, given the conflicting results from previous studies using the CBCL. Indeed, Prosperi et al. (14) reported a higher level of internalizing behaviors in boys at preschool age, whereas Frazier et al. (8) reported a higher level of externalizing behaviors in women at adulthood, and Nasca et al. (28) did not find any sex-related clinical differences in 6- to 12-year-old children. In our sample, the girls had higher severity scores on both the SRS-2 social deficit scale and the CBCL anxiety problem scale, suggesting that their greater social deficit contributes to their increased anxiety, which may represent a selection bias. However, consistent with our results of a high level of internalizing behaviors in girls with ASD, a number of authors have also shown that girls with ASD tend to be first diagnosed with another disorder, such as anxiety disorder (43).

This study had several limitations that limit the generalization of the results. First, there was a limited number of girls in our sample, despite recruitment from the large sample of the ELENA cohort, which may raise issues in generalizing the results given that in our sample the girls have a higher severity of ASD symptoms measured with SRS-2. Second, some of the clinical data used for clinical comparisons according to sex were collected from parental self-questionnaires, such as the SRS-2, the ABC, and the CBCL, and not from direct clinical measurements. Third, there was no control group of typically developing children to verify that clinical differences were not found between the same populations of typically developing children. Fourth, part of the data collection is based on self-administered questionnaires such as the ABC and CBCL completed by parents online and not by direct measurement, which may constitute a bias and may have increased missing data. Finally, our cross-sectional study requires additional further longitudinal analyses, as it is known that individuals with ASD undergo large developmental changes.

In conclusion, our results obtained from a subsample of the ELENA cohort confirm the higher rates of boys with ASD without ID. A comparison between the girls and boys in our study showed them to have similar clinical profiles, except for the SRS- 2 total scores, which were higher among girls, suggesting more severe social impairment perceived by parents. Our findings that the cognitive level is related to ASD severity in girls should be taken into account during the diagnostic procedure in the clinical interpretation of gold-standard measures of ASD, and additional clinical observations are necessary. Our finding that girls with normal intellectual level have more internalized behaviors such as anxiety should lead to awareness in such diagnoses that may mask signs of ASD and delay diagnosis. Clinicians should be aware of this risk and investigate clinically for signs of ASD in girls with atypical relationship impairments attributed to anxiety. Moreover, longitudinal studies are needed to explore sex-related differences in the developmental trajectories in ASD to analyze the course of symptoms according to sex and improve their identification and diagnosis.

## Data availability statement

The original contributions presented in this study are included in the article/Supplementary material, further inquiries can be directed to the corresponding author.

## Ethics statement

The studies involving human participants were reviewed and approved by the Ethics Committee on the Research of Human Subjects at Marseille Mediterranean. Written informed consent to participate in this study was provided by the participants’ legal guardian/next of kin.

## Author contributions

FD and AB conceived the study, contributed to the collection, analysis, interpretation of the data, and drafted the manuscript. AB was the PI of the ELENA cohort. CM, CR, and M-CP analyzed and interpreted the data and critically revised it for the principal intellectual content. All authors read and approved the final version.

## References

[1] Lyall K Croen L Daniels J Fallin MD Ladd-Acosta C Lee BK The changing epidemiology of autism spectrum disorders. *Annu Rev Public Health.* (2017) 38:81–102. 10.1146/annurev-publhealth-031816-044318 28068486 PMC6566093

[2] Maenner M Shaw K Bakian A. Prevalence and characteristics of autism spectrum disorder among children aged 8 years – autism and developmental disabilities monitoring network, 11 Sites, United States, 2018. *MMWR Surveill Summ.* (2021) 70:1–16. 10.15585/mmwr.ss7011a1 PMC8639024 34855725

[3] Fombonne E. Epidemiology of pervasive developmental disorders. *Pediatr Res.* (2009) 65:591. 10.1203/PDR.0b013e31819e7203 19218885

[4] Dworzynski K Ronald A Bolton P Happé F. How different are girls and boys above and below the diagnostic threshold for autism spectrum disorders? *J Am Acad Child Adolesc Psychiatry.* (2012) 51:788–97. 10.1016/j.jaac.2012.05.018 22840550

[5] Idring S Lundberg M Sturm H Dalman C Gumpert C Rai D Changes in prevalence of autism spectrum disorders in 2001–2011: findings from the Stockholm youth cohort. *J Autism Dev Disord.* (2015) 45:1766–73. 10.1007/s10803-014-2336-y 25475364

[6] Werling DM Geschwind DH. Sex differences in autism spectrum disorders. *Curr Opin Neurol.* (2013) 26:146–53. 10.1097/WCO.0b013e32835ee548 23406909 PMC4164392

[7] Xu G Strathearn L Liu B Bao W. Prevalence of autism spectrum disorder among US children and adolescents, 2014–2016. *JAMA.* (2018) 319:81–2. 10.1001/jama.2017.17812 29297068 PMC5833544

[8] Frazier TW Georgiades S Bishop SL Hardan AY. Behavioral and cognitive characteristics of females and males with autism in the Simons Simplex Collection. *J Am Acad Child Adolesc Psychiatry.* (2014) 53:329–40.e1–3. 10.1016/j.jaac.2013.12.004 24565360 PMC3935179

[9] Rutherford M McKenzie K Johnson T Catchpole C O’Hare A McClure I Gender ratio in a clinical population sample, age of diagnosis and duration of assessment in children and adults with autism spectrum disorder. *Autism.* (2016) 20:628–34. 10.1177/1362361315617879 26825959

[10] Loomes R Hull L Mandy WPL. What is the male-to-female ratio in autism spectrum disorder? A systematic review and meta-analysis. *J Am Acad Child Adolesc Psychiatry.* (2017) 56:466–74. 10.1016/j.jaac.2017.03.013 28545751

[11] Rutter M Caspi A Moffitt TE. Using sex differences in psychopathology to study causal mechanisms: unifying issues and research strategies. *J Child Psychol Psychiatry.* (2003) 44:1092–115. 10.1111/1469-7610.00194 14626453

[12] Lai MC Lombardo MV Pasco G Ruigrok ANV Wheelwright SJ Sadek SA A behavioral comparison of male and female adults with high functioning autism spectrum conditions. *PLoS One.* (2011) 6:e20835. 10.1371/journal.pone.0020835 21695147 PMC3113855

[13] Hull L Petrides KV Allison C Smith P Baron-Cohen S Lai MC “Putting on My Best Normal”: social camouflaging in adults with autism spectrum conditions. *J Autism Dev Disord.* (2017) 47:2519–34. 10.1007/s10803-017-3166-5 28527095 PMC5509825

[14] Prosperi M Turi M Guerrera S Napoli E Tancredi R Igliozzi R Sex differences in autism spectrum disorder: an investigation on core symptoms and psychiatric comorbidity in preschoolers. *Front Integr Neurosci.* (2020) 14:594082. 10.3389/fnint.2020.594082 33584212 PMC7876072

[15] Rodgers JD Lodi-Smith J Donnelly JP Lopata C McDonald CA Thomeer ML Brief report: examination of sex-based differences in ASD symptom severity among high-functioning children with ASD using the SRS-2. *J Autism Dev Disord.* (2019) 49:781–7. 10.1007/s10803-018-3733-4 30151783

[16] Ratto AB Kenworthy L Yerys BE Bascom J Wieckowski AT White SW What about the girls? Sex-based differences in autistic traits and adaptive skills. *J Autism Dev Disord.* (2018) 48:1698–711. 10.1007/s10803-017-3413-9 29204929 PMC5925757

[17] Van Wijngaarden-Cremers PJM van Eeten E Groen WB Van Deurzen PA Oosterling IJ Van der Gaag RJ. Gender and age differences in the core triad of impairments in autism spectrum disorders: a systematic review and meta-analysis. *J Autism Dev Disord.* (2014) 44:627–35. 10.1007/s10803-013-1913-9 23989936

[18] Lawson LP. Sex differences in autism spectrum disorders across the lifespan. *Curr Dev Disord Rep.* (2019) 6:57–66. 10.1007/s40474-019-00164-y

[19] de Giambattista C Ventura P Trerotoli P Margari F Margari L. Sex differences in autism spectrum disorder: focus on high functioning children and adolescents. *Front Psychiatry.* (2021) 12:539835. 10.3389/fpsyt.2021.539835 34305658 PMC8298903

[20] Siracusano M Postorino V Riccioni A Emberti Gialloreti L Terribili M Curatolo P Sex differences in autism spectrum disorder: repetitive behaviors and adaptive functioning. *Children.* (2021) 8:325. 10.3390/children8050325 33922236 PMC8146768

[21] Knutsen J Crossman M Perrin J Shui A Kuhlthau K. Sex differences in restricted repetitive behaviors and interests in children with autism spectrum disorder: an autism treatment network study. *Autism.* (2019) 23:858–68. 10.1177/1362361318786490 30047281 PMC6348057

[22] Osório JMA Rodríguez-Herreros B Richetin S Junod V Romascano D Pittet V Sex differences in sensory processing in children with autism spectrum disorder. *Autism Res.* (2021) 14:2412–23. 10.1002/aur.2580 34288517 PMC9290069

[23] Duvall SW Huang-Storms L Presmanes Hill A Myers J Fombonne E. No sex differences in cognitive ability in young children with autism spectrum disorder. *J Autism Dev Disord.* (2020) 50:1770–85. 10.1007/s10803-019-03933-1 30810843

[24] Mahendiran T Dupuis A Crosbie J Georgiades S Kelley E Liu X Sex differences in social adaptive function in autism spectrum disorder and attention-deficit hyperactivity disorder. *Front Psychiatry.* (2019) 10:607. 10.3389/fpsyt.2019.00607 31572228 PMC6751776

[25] Oakland T Harrison PL. Preface. In: Oakland T Harrison PL editors. *Adaptive Behavior Assessment System-II [Internet].* San Diego, CA: Academic Press (2008). p. 19–20. 10.1016/B978-012373586-7.00001-1

[26] McQuaid GA Pelphrey KA Bookheimer SY Dapretto M Webb SJ Bernier RA The gap between IQ and adaptive functioning in autism spectrum disorder: disentangling diagnostic and sex differences. *Autism.* (2021) 25:1565–79. 10.1177/1362361321995620 33715473 PMC8324508

[27] Reynolds CR Kamphaus RW. *Behavior Assessment System for Children.* 2nd ed. Circle Pines, MN: AGS (2004).

[28] Nasca BC Lopata C Donnelly JP Rodgers JD Thomeer ML. Sex differences in externalizing and internalizing symptoms of children with ASD. *J Autism Dev Disord.* (2020) 50:3245–52. 10.1007/s10803-019-04132-8 31278524

[29] Baghdadli A Miot S Rattaz C Akbaraly T Geoffray MM Michelon C Investigating the natural history and prognostic factors of ASD in children: the multicEntric Longitudinal study of childrEN with ASD – the ELENA study protocol. *BMJ Open.* (2019) 9:e026286. 10.1136/bmjopen-2018-026286 31221874 PMC6588969

[30] Wechsler D. *WISC-V: Administration and Scoring Manual.* San Antonio, TX: NCS Pearson, Incorporated (2014).

[31] Wechsler D. *WPPSI-IV, Échelle D’intelligence de Wechsler Pour Enfants.* Paris: ECPA (2014).

[32] Wechsler D. *Wechsler Intelligence Scale for Children-WISC-IV.* San Antonio, TX: Psychological Corporation (2003). 10.1037/t15174-000

[33] Wechsler D. *Wechsler Preschool and Primary Scale of Intelligence™ – Third Edition (WPPSI™ – III).* San Antonio, TX: The Psychological Corporation (2002). 10.1037/t15177-000

[34] Kaufman A Kaufman N. *Kaufman Assessment Battery for Children: Technical Manual.* 2nd ed. Circle Pines, MN: American Guidance Service (2004).

[35] Constantino JN Gruber CP. *The Social Responsiveness Scale™, Second Edition (SRS-2).* Torrance, CA: Western Psychological Services (2012).

[36] Lord C Rutter M Di Lavore P Risi S Gotham K Bishop S. *Autism Diagnostic Observation Schedule, Second Edition (ADOS-2) Manual (Part I): Modules 1–4.* Torrance, CA: Western Psychological Services (2012).

[37] Le Couteur A Lord C Rutter M. *Autism Diagnostic Interview, Revised (ADI-R).* Los Angeles, CA: Western Psychological Services (2003).

[38] Dunn W. *The Sensory Profile: User’s Manual.* San Antonio, TX: Psychological Corporation (1999). 146 p.

[39] Sparrow SS Cicchetti DV Balla DA. *Vineland Adaptive Behavior Scales.* 2nd ed. Circle Pines, MN: AGS Publishing (2005). 10.1037/t15164-000

[40] Aman MG Singh NN Stewart AW Field CJ. The aberrant behavior checklist: a behavior rating scale for the assessment of treatment effects. *Am J Ment Defic.* (1985) 89:485–91. 10.1037/t10453-000 3993694

[41] Achenbach TM. *Achenbach System of Empirically Based Assessment (ASEBA): Development, Findings, Theory, and Applications.* Burlington: University of Vermont Research Center for Children, Youth, and Families (2009). 154 p.

[42] Halladay AK Bishop S Constantino JN Daniels AM Koenig K Palmer K Sex and gender differences in autism spectrum disorder: summarizing evidence gaps and identifying emerging areas of priority. *Mol Autism.* (2015) 6:36. 10.1186/s13229-015-0019-y 26075049 PMC4465158

[43] Rødgaard EM Jensen K Miskowiak KW Mottron L. Autism comorbidities show elevated female-to-male odds ratios and are associated with the age of first autism diagnosis. *Acta Psychiatr Scand.* (2021) 144:475–86. 10.1111/acps.13345 34228813 PMC9292172

