# Supplementary material for: Sex-related differences in clinical characteristics of children with ASD without ID: Results from the ELENA cohort
Source: Front Psychiatry. 2022 Nov 28;13:998195. doi: 10.3389/fpsyt.2022.998195 (PMC9742240; doi:10.3389/fpsyt.2022.998195)
Supplement: Supplementary file 1 [file Table_1.docx]

Supplementary Table S1: Coefficients of correlation between SRS-2 score and clinical characteristics for girls

|  | **SRS-2** | | | | | | | | | | | | | |
| --- | --- | --- | --- | --- | --- | --- | --- | --- | --- | --- | --- | --- | --- | --- |
|  | **Social awareness** | | **Social cognition** | | **Social communication** | | **Social motivation** | | **RIRB** | | **SCI** | | **Total score** | |
| **Age** | -0.08 36 | | -0.27 34 | | 0.11 33 | | -0.029 35 | | 0.037 35 | | -0.12 31 | | 0.076 36 | |
| **IQ** | -0.036 36 | | **-0.51* 34** | | -0.23 33 | | **-0.39* 35** | | **-0.40* 35** | | **-0.43* 31** | | **-0.37* 36** | |
| **Age first diagnosis** | -0.04 33 | | -0.31 31 | | 0.06 30 | | 0.016 32 | | 0.01 33 | | -0.08 28 | | 0.07 33 | |
| **Age first psychiatric advice** | -0.07 33 | | -0.20 31 | | 0.22 30 | | 0.17 32 | | 0.03 33 | | 0.02 28 | | 0.10 33 | |
| **ADOS severity score** | 0.16 34 | | 0.1 32 | | 0.002 31 | | -0.05 33 | | 0.05 33 | | 0.2 29 | | 0.02 34 | |
| **ADI-R-** Communication verbal | 0.02 29 | | 0.30 28 | | -0.16 27 | | 0.21 28 | | -0.11 30 | | 0.15 25 | | 0.02 30 | |
| **ADI-R-** Communication non verbal | 0.21 12 | | 0.23 11 | | -0.06 11 | | 0.15 12 | | 0.130 11 | | 0.2 11 | | 0.26 11 | |
| **ADI-R** - Social reciprocity | 0.13 33 | | 0.29 31 | | 0.056 30 | | 0.17 32 | | 0.23 33 | | 0.18 28 | | 0.23 33 | |
| **ADI-R** -Restricted and repetitive behaviors | 0.29 33 | | 0.06 31 | | 0.08 30 | | 0.34 32 | | -0.03 33 | | 0.23 28 | | 0.08 33 | |
| **VABS-II-** Communication | -0.15 36 | | -0.27 34 | | -0.17 33 | | -0.15 35 | | -0.25 35 | | -0.22 31 | | -0.27 36 | |
| **VABS-II-** Socialization | -0.25 36 | | -0.10 34 | | -0.17 33 | | -0.15 35 | | -0.30 35 | | -0.12 31 | | -0.29 36 | |
| **VABS-II-** Daily living skills | **-0.43* 36** | | -0.16 34 | | -0.23 33 | | -0.20 35 | | -0.27 35 | | -0.26 31 | | -0.29 36 | |
| **Sensory Profile-** Short Total Score | -0.27 27 | | **-0.49* 25** | | **-0.45* 24** | | **-0.42* 26** | | -0.29 27 | | **-0.46* 22** | | **-0.39* 27** | |
| **ABC-** Irritability, uncooperative | **0.40* 29** | | **0.45* 28** | | 0.35 27 | | 0.29 28 | | 0.36 28 | | 0.40 25 | | **0.42* 29** | |
| **ABC-** Lethargy, withdrawal | **0.38* 29** | | **0.61** 28** | | **0.70*** 27** | | **0.57** 28** | | **0.62** 28** | | **0.70*** 25** | | **0.73*** 29** | |
| **ABC**- Stereotypy | **0.42* 29** | | **0.60** 28** | | **0.57* 27** | | 0.24 28 | | **0.66** 28** | | **0.52** 25** | | **0.60** 29** | |
| **ABC-** Hyperactivity | 0.20 30 | | 0.21 29 | | 0.261 28 | | 0.016 29 | | 0.20774 29 | | 0.13 26 | | 0.18 30 | |
|  |  | | | | | | | | | | | | | |
| **CBCL-Internal** | **N** | **M ± SD** | **N** | **M ± SD** | **N** | **M ± SD** | **N** | **M ± SD** | **N** | **M ± SD** | **N** | **M ± SD** | **N** | **M ± SD** |
| normal | 7 | **67.4±15.4*** | 6 | 81.8±23.2 | 7 | **78.7 ± 16.1*** | 6 | **60.8±17.2*** | 7 | 100.7±34.7 | 5 | **78.0± 22.9*** | 7 | **81.6± 22.1*** |
| clinical | 23 | **82.1±12.9*** | 23 | 96.1±13.4 | 21 | **95.6 ± 12.2*** | 23 | **80.0±11.5*** | 22 | 119.6±22.6 | 21 | **96.5± 12.0*** | 23 | **103.2± 13.5*** |
| **CBCL-External** |  |  |  |  |  |  |  |  |  |  |  |  |  |  |
| normal | 13 | **71.1±14.5*** | 13 | 88.8±17.3 | 12 | 85.7±16.6 | 12 | 72.2±16.7 | 14 | 106.9±29.6 | 10 | 87.4±18.6 | 14 | 92.1±19.6 |
| clinical | 17 | **84.5±12.3*** | 16 | 96.8±15.4 | 16 | 95.6±12.3 | 17 | 78.8±13.1 | 15 | 122.7±21.7 | 16 | 96.4±13.5 | 16 | 103.4±15.2 |
| **CBCL-Affective problem** | |  |  |  |  |  |  |  |  |  |  |  |  |  |
| normal | 11 | 72.3±15.4 | 9 | 88.8±21.5 | 10 | 85.2±17.9 | 10 | 71.2±20.0 | 10 | 111.0±35.3 | 8 | 87.3±22.4 | 10 | 90.4±24.0 |
| clinical | 19 | 82.4±13.9 | 20 | 95.2±13.9 | 18 | 94.8±12.2 | 19 | 78.6±11.0 | 19 | 117.2±21.5 | 18 | 95.4±12.0 | 20 | 102.0±13.3 |
| **CBCL-Anxiety problem** | |  |  |  |  |  |  |  |  |  |  |  |  |  |
| normal | 9 | 76.4±19.9 | 7 | 85.4±23.9 | 8 | 82.9±18.2 | 8 | **66.8±19.2*** | 8 | 105.3±33.6 | 6 | 84.8±25.5 | 8 | **87.1±25.4*** |
| clinical | 21 | 79.7±12.3 | 22 | 95.6±13.1 | 20 | 94.7±12.4 | 21 | **79.6±11.4*** | 21 | 118.8±23.2 | 20 | 95.4±11.7 | 22 | **102.1±13.1*** |
| **CBCL-Attention deficit** | |  |  |  |  |  |  |  |  |  |  |  |  |  |
| normal | 14 | 76.6±17.0 | 13 | 93.3±19.6 | 13 | 90.0±17.9 | 13 | 76.9±18.1 | 14 | 113.5±30.7 | 12 | 92.8±19.4 | 14 | 97.1±21.7 |
| clinical | 16 | 80.5±12.6 | 16 | 93.1±14.2 | 15 | 92.5±12.3 | 16 | 75.4±12.1 | 15 | 116.5±23.1 | 14 | 93.1±12.9 | 16 | 99.1±14.7 |
| **CBCL-Oppositional problem** | |  |  |  |  |  |  |  |  |  |  |  |  |  |
| normal | 20 | 75.6±13.9 | 19 | 89.8±17.4 | 18 | **86.9±14.2*** | 19 | **72.1±14.1*** | 20 | 109.6±27.2 | 16 | 88.5±15.7 | 20 | 93.5±17.6 |
| clinical | 10 | 85.0±15.0 | 10 | 99.5±12.9 | 10 | **99.3±13.4*** | 10 | **83.5±13.7*** | 9 | 127.1±21.9 | 10 | 100.0±14.1 | 10 | 107.4±15.8 |
| **CBCL-Somatic problem** |  |  |  |  |  |  |  |  |  |  |  |  |  |  |
| normal | 20 | 79.5±11.9 | 21 | 94.7±13.2 | 20 | 91.9±11.4 | 20 | 77.2±11.2 | 20 | 116.7±22.5 | 19 | 93.4±11.9 | 21 | 99.4±13.9 |
| clinical | 4 | 79.0±14.4 | 4 | 90.3±13.2 | 3 | 103.3±11.2 | 3 | 83.0±11.8 | 4 | 122.5±21.7 | 3 | 99.0±9.2 | 4 | 104.8±9.0 |
| **CBCL-Conduct problem** |  |  |  |  |  |  |  |  |  |  |  |  |  |  |
| normal | 17 | 79.4±13.1 | 18 | 96.8±11.6 | 16 | 94.1±11.3 | 16 | 78.5±8.7 | 18 | 120.5±22.4 | 15 | 95.8±9.7 | 18 | 101.7±12.1 |
| clinical | 7 | 79.4±9.9 | 7 | 88.6±14.4 | 7 | 91.9±13.7 | 7 | 76.6±16.5 | 6 | 109.2±20.1 | 7 | 90.6±15.0 | 7 | 96.4±16.3 |
| **CBCL-Pervasive problem** | |  |  |  |  |  |  |  |  |  |  |  |  |  |
| normal | 2 | - | 1 | - | 2 | - | 2 | - | 2 | - | 1 | - | 2 | - |
| clinical | 4 | - | 3 | - | 3 | - | 4 | - | 3 | - | 3 | - | 3 | - |

^β^:  ρ : Spearman’s Coefficient correlation and N=sample size;

Significant associations (p-value<.05) are presented in bold;

P-value is marked: *<.05; **<.001; ***<.0001;
