# Supplementary material for: Sex-related differences in clinical characteristics of children with ASD without ID: Results from the ELENA cohort
Source: Front Psychiatry. 2022 Nov 28;13:998195. doi: 10.3389/fpsyt.2022.998195 (PMC9742240; doi:10.3389/fpsyt.2022.998195)
Supplement: Supplementary file 2 [file Table_2.docx]

Supplementary Table S2: Coefficients of correlation between SRS-2 score and clinical characteristics for boys

|  | **SRS-2** | | | | | | | | | | | | | |
| --- | --- | --- | --- | --- | --- | --- | --- | --- | --- | --- | --- | --- | --- | --- |
|  | **Social awareness** | | **Social cognition** | | **Social communication** | | **Social motivation** | | **RIRB** | | **SCI** | | **Total score** | |
| **Age** | -0.009^β^ 145 | | 0.1 135 | | -0.08 133 | | 0.1 142 | | 0.09 143 | | -0.075 124 | | -0.04 148 | |
| **IQ** | 0.05 145 | | 0.085 135 | | -0.01 133 | | 0.03 142 | | 0.09 143 | | -0.02 124 | | -0.006 148 | |
| **Age first diagnosis** | -0.07 128 | | 0.07 119 | | -0.17 119 | | 0.03 126 | | 0.03 127 | | -0.15 112 | | -0.07 130 | |
| **Age first psychiatric advice** | **-0.2* 128** | | -0.06 119 | | -0.16 119 | | 0.02 126 | | -0.009 127 | | **-0.18* 112** | | -0.11 130 | |
| **ADOS severity score** | 0.036 128 | | 0.005 123 | | 0.08 121 | | 0.13 127 | | 0.055 127 | | 0.07 114 | | 0.08 131 | |
| **ADI-R-** Communication verbal | **0.37** 96** | | **0.42*** 93** | | **0.45*** 91** | | **0.39*** 96** | | **0.35** 95** | | **0.46*** 86** | | **0.44*** 98** | |
| **ADI-R-** Communication non verbal | **0.29* 44** | | **0.36* 40** | | **0.40* 41** | | 0.23 42 | | 0.13 44 | | **0.36* 39** | | **0.3* 44** | |
| **ADI-R** - Social reciprocity | **0.4*** 127** | | **0.49*** 121** | | **0.52*** 119** | | **0.44*** 125** | | **0.44*** 126** | | **0.54*** 113** | | **0.49*** 129** | |
| **ADI-R** -Restricted and repetitive behaviors | 0.1  127 | | **0.29****  **121** | | **0.34****  **119** | | **0.22***  **125** | | **0.34****  **126** | | **0.31****  **113** | | **0.30****  **129** | |
| **VABS-II-** Communication | **-0.16* 145** | | **-0.23* 135** | | **-0.2* 133** | | **-0.2* 142** | | -0.1 143 | | **-0.25* 124** | | **-0.24* 148** | |
| **VABS-II-** Socialization | **-0.30** 145** | | **-0.45*** 135** | | **-0.35*** 133** | | **-0.43*** 142** | | **-0.31*** 143** | | **-0.42*** 124** | | **-0.40*** 148** | |
| **VABS-II-** Daily living skills | -0.096 145 | | **-0.31** 135** | | -0.10 133 | | **-0.22* 142** | | **-0.20* 143** | | **-0.19* 124** | | **-0.20* 148** | |
| **Sensory Profile-** Short Total Score | **-0.28* 109** | | **-0.43** 101** | | **-0.42*** 100** | | **-0.39*** 107** | | **-0.54*** 107** | | **-0.37** 93** | | **-0.46*** 111** | |
| **ABC-** Irritability, uncooperative | **0.30* 114** | | **0.44*** 107** | | **0.41*** 105** | | **0.42*** 110** | | **0.47*** 112** | | **0.44*** 99** | | **0.46*** 115** | |
| **ABC-** Lethargy, withdrawal | **0.37*** 114** | | **0.51*** 107** | | **0.50*** 105** | | **0.68*** 110** | | **0.42*** 112** | | **0.59*** 99** | | **0.55*** 115** | |
| **ABC**- Stereotypy | **0.2* 114** | | **0.52*** 107** | | **0.49*** 105** | | **0.49*** 110** | | **0.58*** 112** | | **0.51*** 99** | | **0.53*** 115** | |
| **ABC-** Hyperactivity | **0.3* 114** | | **0.47*** 107** | | **0.40*** 105** | | **0.35** 110** | | **0.49*** 112** | | **0.41*** 99** | | **0.46*** 115** | |
|  |  | | | | | | | | | | | | | |
| **CBCL-Internal** | **N** | **M ± SD** | **N** | **M ± SD** | **N** | **M ± SD** | **N** | **M ± SD** | **N** | **M ± SD** | **N** | **M ± SD** | **N** | **M ± SD** |
| normal | 23 | **69.4±15.9**** | 20 | **72.7±18.9***** | 18 | **69.2 ± 18.5***** | 22 | **63.5±18.3 ***** | 22 | **74.2±22.4***** | 15 | **73.5± 19.7***** | 24 | **75.5± 22.1***** |
| clinical | 41 | **80.3±14.1**** | 38 | **91.6±16.0***** | 39 | **95.4 ± 19.2***** | 39 | **82.8±13.9***** | 40 | **102.0±23.4***** | 36 | **96.7± 17.8***** | 41 | **99.4± 18.2***** |
| **CBCL-External** |  |  |  |  |  |  |  |  |  |  |  |  |  |  |
| normal | 45 | 73.1±15.1 | 44 | **78.7±16.0***** | 40 | **80.5±19.5*** | 42 | **71.3±17.6*** | 45 | **83.1±22.0***** | 36 | **83.0±17.5*** | 46 | **82.9±19.2***** |
| clinical | 69 | 78.5±13.7 | 63 | **92.2±15.9***** | 65 | **90.4±15.8*** | 68 | **79.4±14.9*** | 67 | **101.7±18.9***** | 63 | **91.9±16.6*** | 69 | **95.9±16.9***** |
| **CBCL-Affective problem** | |  |  |  |  |  |  |  |  |  |  |  |  |  |
| normal | 47 | 73.6±14.0 | 42 | **77.9±16.6***** | 40 | **78.9±17.7**** | 45 | **67.3±14.2***** | 45 | **82.3±20.7***** | 36 | **80.3±15.8***** | 48 | **81.8±17.9***** |
| clinical | 66 | 78.5±14.5 | 64 | **92.6±15.1***** | 64 | **91.8±16.1**** | 64 | **82.9±14.8***** | 66 | **102.6±19.2***** | 62 | **93.9±16.3***** | 66 | **97.4±16.8***** |
| **CBCL-Anxiety problem** | |  |  |  |  |  |  |  |  |  |  |  |  |  |
| normal | 53 | **72.8±15.6*** | 48 | **80.3±18.4***** | 47 | 84.1±21.1 | 50 | **71.3±17.4*** | 51 | **85.8±24.9***** | 42 | 85.3±20.0 | 54 | **86.0±21.8*** |
| clinical | 60 | **79.7±12.7*** | 58 | **92.2±14.2***** | 57 | 89.1±14.4 | 59 | **80.9±14.3*** | 60 | **101.7±16.4***** | 56 | 91.6±14.6 | 60 | **95.2±14.6*** |
| **CBCL-Attention deficit** | |  |  |  |  |  |  |  |  |  |  |  |  |  |
| normal | 62 | 74.6±15.7 | 59 | **81.8±17.3**** | 58 | 84.2±18.8 | 58 | 74.0±17.9 | 61 | **85.6±21.45***** | 52 | 86.19±18.2 | 63 | **86.4±19.7*** |
| clinical | 51 | 78.7±12.6 | 47 | **93.0±15.2**** | 46 | 90.2±16.2 | 51 | 79.3±14.2 | 50 | **105.1±17.9***** | 46 | 91.91±15.9 | 51 | **96.4±16.3*** |
| **CBCL-Oppositional problem** | |  |  |  |  |  |  |  |  |  |  |  |  |  |
| normal | 84 | 74.9±14.5 | 78 | **84.5±17.4*** | 76 | 84.87±18.55 | 80 | **73.9±16.9*** | 82 | **90.2±22.1**** | 70 | 86.6±17.7 | **85** | **88.0±19.2*** |
| clinical | 30 | 80.3±13.7 | 29 | **92.6±15.4*** | 29 | 91.28±15.28 | 30 | **82.8±13.2*** | 30 | **105.2±18.3**** | 29 | 93.7±15.7 | **30** | **98.3±16.2*** |
| **CBCL-Somatic problem** |  |  |  |  |  |  |  |  |  |  |  |  |  |  |
| normal | 42 | 75.5±14.4 | 41 | 86.2±14.7 | 39 | 84.3±9.9 | 40 | 73.8±14.2 | 41 | **93.5±14.2*** | 38 | 85.6±11.8 | 42 | 87.8±13.0 |
| clinical | 13 | 76.9±13.7 | 13 | 92.6±14.8 | 13 | 89.8±12.1 | 13 | 82.3±17.4 | 13 | **104.0±13.6*** | 13 | 91.6±14.6 | 13 | 95.6±14.2 |
| **CBCL-Conduct problem** |  |  |  |  |  |  |  |  |  |  |  |  |  |  |
| normal | 39 | 76.1±14.1 | 38 | 87.2±14.6 | 36 | 86.2±11.6 | 37 | 75.7±16.2 | 38 | 95.4±15.0 | 35 | 87.5±13.1 | 39 | 89.5±14.2 |
| clinical | 16 | 75.4±14.6 | 16 | 88.9±15.8 | 16 | 84.4±8.5 | 16 | 76.4±13.5 | 16 | 97.6±13.9 | 16 | 86.4±12.3 | 16 | 90.0±12.5 |
| **CBCL-Pervasive problem** | |  |  |  |  |  |  |  |  |  |  |  |  |  |
| normal | 15 | **65.3±12.7***** | 14 | **67.4±15.2***** | 14 | **63.4±14.1***** | 15 | **57.9±12.1***** | 15 | **66.0±18.0***** | 12 | **68.0±13.5***** | 16 | **66.9±14.8***** |
| clinical | 43 | **81.1±13.5***** | 38 | **92.6±16.4***** | 38 | **97.1±18.5***** | 41 | **83.9±13.6***** | 42 | **102.4±23.7***** | 35 | **98.6±17.5***** | 43 | **101.4±17.6***** |

^β^:  ρ : Spearman’s Coefficient correlation and N=sample size;

Significant associations (p-value<.05) are presented in bold; P-value is marked: *<.05; **<.001; ***<.0001;
